# Supplementary material for: Preparing future STEM faculty through flexible teaching professional development
Source: PLoS One. 2023 Oct 12;18(10):e0276349. doi: 10.1371/journal.pone.0276349 (PMC10569627; doi:10.1371/journal.pone.0276349)
Supplement: S1 File — (DOCX) [file pone.0276349.s001.docx]

**Preparing future STEM faculty through flexible teaching professional development**

# **Supporting materials: Analysis of course participation, activity, and outcomes**

[**Supporting materials: Analysis of course participation, activity, and outcomes**](#_heading=h.30j0zll) **1**

[Course instances](#_heading=h.1fob9te) 1

[Data Sources](#_heading=h.e7vhhce24wmt) 2

[Survey participant demographics](#_heading=h.2et92p0) 3

[Participant course activity](#_heading=h.tyjcwt) 7

[Definition of course participant](#_heading=h.3dy6vkm) 8

[Quizzes](#_heading=h.1t3h5sf) 9

[Videos](#_heading=h.4d34og8) 9

[Peer-graded assessments](#_heading=h.2s8eyo1) 10

[Learners](#_heading=h.17dp8vu) 10

[Course completion](#_heading=h.3rdcrjn) 12

[Course activity of survey respondents](#_heading=h.26in1rg) 13

[Motivations for taking the course](#_heading=h.lnxbz9) 15

[Helpfulness of course components](#_heading=h.35nkun2) 16

[Learning indicators](#_heading=h.1ksv4uv) 17

[Learning community/MCLC engagement](#_heading=h.44sinio) 18

[MCLC participation](#_heading=h.2jxsxqh) 18

[Motivation for MCLC participation](#_heading=h.z337ya) 19

## **Course instances**

Two MOOCs were created and offered. An introductory course, *An Introduction to Evidence-based Undergraduate STEM Learning,* and an advanced course, *Advanced Learning Through Evidence-Based STEM Teaching.* The introductory course was offered seven times from 2014 through 2018. The advanced course was offered four times from 2016 through 2018.

As noted in the paper, based on feedback from MOOC-Centered Learning Community (MCLC) facilitators and participants, the instructors significantly revised the content and structure of the advanced course between the third and fourth instances. Because of this change, plus the relatively smaller number of participants in the advanced course, the analysis in the paper and below focuses only on the seven instances of the introductory course.

## **Data sources**

As discussed in the manuscript, there are four main sources of data on course participation, engagement, and outcomes. Briefly, these are course enrollment, engagement, activity, and completion information from the online platforms; pre-course and post-course surveys; and MCLC participant surveys.

## **Survey participant demographics**

We examined who took the pre- and post-course surveys. While there are significantly higher numbers in the first two instances of the course, there is consistency of response rate across courses and across all demographic categories for both pre- and post-survey respondents. These results give us confidence that while the course audience has become smaller over time, it has not significantly changed in make-up. S1 and S2 Tables display the enrollment data, rate of pre- and post-survey response, demographics of participants, location in a CIRTL institution, status or role, teaching preparation as motivation, intention to participate in an MCLC (pre-survey), actual participation in an MCLC (post-survey), and academic discipline.

**S1 Table. Enrollment and demographics from 2014-2018 in *An Introduction to Evidence-based Undergraduate STEM Learning.***

|  | | **Fall 2014** | **Fall 2015** | **Fall 2016** | **Fall 2017** | **Spring 2017** | **Summer 2017** | **Summer 2018** | **Average**** |
| --- | --- | --- | --- | --- | --- | --- | --- | --- | --- |
| **Total enrolled** | | 5909 | 3822 | 1449 | 894 | 978 | 935 | 990 | 2139 |
| **Pre-survey respondents** | | 1418 | 869 | 476 | 183 | 261 | 327 | 350 | 554 |
| **Percent of enrolled users completing pre-survey** | | 24% | 23% | 33% | 20% | 27% | 35% | 35% | 28% |
| **Countries represented** | | 75 | 55 | 47 | 20 | 37 | 27 | 46 | 43 |
| **CIRTL Institution** | | 29.1% | 26.7% | 40.5% | 48.6% | 36.8% | 50.5% | 39.1% | 38.8% |
| **Role*** | |  |  |  |  |  |  |  |  |
|  | Doctoral student | 28.5% | 30.3% | 27.3% | 32.2% | 21.1% | 32.7% | 29.1% | 28.7% |
|  | Post-doctoral researcher | 18.3% | 19.0% | 20.0% | 26.8% | 28.4% | 25.4% | 21.1% | 22.7% |
|  | Faculty | 20.1% | 24.6% | 13.9% | 25.1% | 15.3% | 17.4% | 19.4% | 19.4% |
|  | Other | 32.8% | 25.5% | 34.2% | 15.8% | 31.8% | 23.2% | 30.0% | 27.6% |
| **Gender*** | |  |  |  |  |  |  |  |  |
|  | Female | 60.3% | 63.2% | 54.4% | 62.8% | 61.3% | 60.9% | 56.6% | 59.9% |
|  | Male | 37.2% | 34.8% | 32.6% | 31.7% | 36.4% | 35.5% | 42.3% | 35.8% |
| **Preparing to teach*** | |  |  |  |  |  |  |  |  |
|  | True | 80.6% | 63.4% | 64.1% | 68.9% | 67.8% | 71.6% | 69.4% | 69.4% |
|  | False | 19.4% | 36.6% | 35.9% | 31.1% | 32.2% | 28.4% | 30.6% | 30.6% |
| **Plan to participate in MCLC*** | |  |  |  |  |  |  |  |  |
|  | Yes | 29.5% | 31.0% | 20.6% | 30.1% | 25.3% | 22.3% | 24.3% | 26.1% |
|  | No | 27.3% | 24.1% | 22.3% | 19.7% | 30.7% | 30.9% | 25.1% | 25.7% |
|  | I'm not sure | 42.5% | 43.8% | 44.5% | 50.3% | 42.5% | 45.6% | 49.7% | 45.6% |
| **STEM/SBE field** | | 93.3% | 91.5% | 85.1% | 89.6% | 87.0% | 88.1% | 91.7% | 89.5% |
| **Academic field** | |  |  |  |  |  |  |  |  |
|  | Biological sciences | 39.3% | 41.5% | 38.4% | 45.9% | 38.7% | 39.4% | 36.9% | 40.0% |
|  | Physical sciences | 19.5% | 17.5% | 14.9% | 18.0% | 11.1% | 15.3% | 17.1% | 16.2% |
|  | Engineering | 12.1% | 11.0% | 10.3% | 11.5% | 11.1% | 12.8% | 14.9% | 12.0% |
|  | Other | 29.1% | 29.9% | 33.8% | 24.6% | 38.3% | 31.8% | 30.9% | 31.2% |

S1 Table Legend: Enrollment data, rate of pre-survey response, demographics of participants, location in a CIRTL institution, status or role, teaching preparation as motivation, intention to participate in an MCLC (pre-survey) and academic discipline.

*Given as percentages of pre-course survey respondents

**Averaged over course instances. Not weighted by the number of students per instance.

**S2 Table. Post-course respondent demographics from 2014-2018 in *An Introduction to Evidence-based Undergraduate STEM Learning.***

|  | | **Fall 2014** | **Fall 2015** | **Fall 2016** | **Fall 2017** | **Spring 2017** | **Summer 2017** | **Summer 2018** | **Average**** |
| --- | --- | --- | --- | --- | --- | --- | --- | --- | --- |
| **Post-survey respondents** | | 446 | 186 | 143 | 70 | 59 | 95 | 100 | 157 |
| **CIRTL Institution** | | 28.0% | 37.6% | 50.3% | 58.6% | 33.9% | 58.9% | 50.0% | 45.3% |
| **Role*** | |  |  |  |  |  |  |  |  |
|  | Doctoral student | 23.5% | 29.6% | 37.1% | 30.0% | 22.0% | 37.9% | 38.0% | 31.2% |
|  | Post-doctoral researcher | 18.6% | 29.0% | 21.0% | 37.1% | 45.8% | 25.3% | 22.0% | 28.4% |
|  | Faculty | 16.1% | 23.7% | 15.4% | 22.9% | 3.4% | 14.7% | 16.0% | 16.0% |
|  | Other | 22.4% | 17.2% | 18.2% | 8.6% | 27.1% | 21.1% | 19.0% | 19.1% |
| **Gender*** | |  |  |  |  |  |  |  |  |
|  | Female | 49.1% |  | 51.7% | 55.7% | 64.4% | 65.3% | 48.0% | 55.7% |
|  | Male | 30.5% |  | 35.7% | 35.7% | 30.5% | 32.6% | 45.0% | 35.0% |
| **Ethnicity** | |  |  |  |  |  |  |  |  |
|  | Caucasian (non-Hispanic) |  |  | 51.7% | 62.9% | 62.7% | 66.3% | 45.0% | 57.7% |
|  | Asian/Pacific Islander |  |  | 14.0% | 8.6% | 16.9% | 14.7% | 20.0% | 14.8% |
|  | Other/Multiple |  |  | 25.2% | 22.9% | 16.9% | 13.7% | 27.0% | 21.1% |
| **Participated in MCLC*** | |  |  |  |  |  |  |  |  |
|  | yes | 32.5% | 35.5% | 32.2% | 30.0% | 25.4% | 30.5% | 39.0% | 32.2% |
|  | no | 63.9% | 62.4% | 62.2% | 70.0% | 72.9% | 69.5% | 59.0% | 65.7% |
| **Citizenship* ***** | |  |  |  |  |  |  |  |  |
|  | U.S. citizen or permanent resident |  |  | 63.6% | 71.4% | 55.9% | 80.0% | 61.0% | 66.4% |
|  | Other non-U.S. citizen |  |  | 27.3% | 22.9% | 40.7% | 18.9% | 32.0% | 28.4% |

S2 Table Legend: Enrollment data, rate of post-survey response, demographics of participants, location in a CIRTL institution, status or role, actual participation in an MCLC (post-survey) and academic discipline.

*Given as percentages of post-course survey respondents.

**Averaged over course instances. Not weighted by number of students per instance.

***This question was added starting with the third iteration of the course.

## **Participant course activity**

We examined four activities participants could engage in during the course: taking quizzes, completing PGAs, watching course videos, and participating in an MCLC. The last activity is not well documented, because we were not allowed to collect names of MCLC participants and associate them with their activity in the online course (IRB restriction). We did not analyze activity on the discussion forums because threads tended to be relatively short compared to the numbers of students watching videos or completing quizzes. Minimal analysis of discussion participants from the edX analytics did not reveal any surprising results. Additional optional learning materials and links are provided in the course modules, but as the information is not central to the course, we did not analyze participant utilization.

### **Definition of course participant**

Across all instances of the courses, 48% of those enrolled do at least one of the following activities in the course: (1) Attempt a quiz; (2) Watch a video (specifically open the video and start playing it); or (3) Submit a PGA. For the purposes of choosing a relevant baseline comparison group for the analyses below, we call the 48% of those who did an activity in the course, ‘course participants.’ The remaining 52% enrolled in the course but did not engage with the material in any significant way.

### **Quizzes**

Each module has a quiz with 5-8 questions based on the material covered in the course videos, readings and discussion prompts. A majority of participants either completed no quizzes or all of the quizzes (see Fig. S1 (a)). 66% percent of participants who completed at least two ended up completing all six.


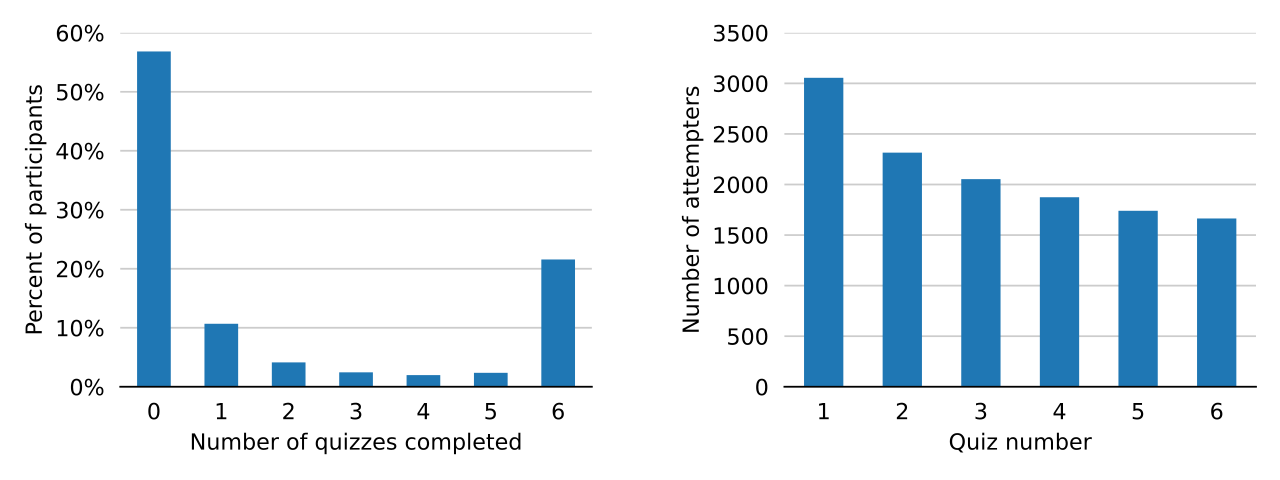


**S1 Fig. Quiz completion.** (A) Histogram of total number of quizzes completed. (B) Number of participants who completed each quiz.

### **Videos**

There are 108 videos over the six course modules in the introductory course. Each module has an introductory video, a concluding video and multiple topical videos.


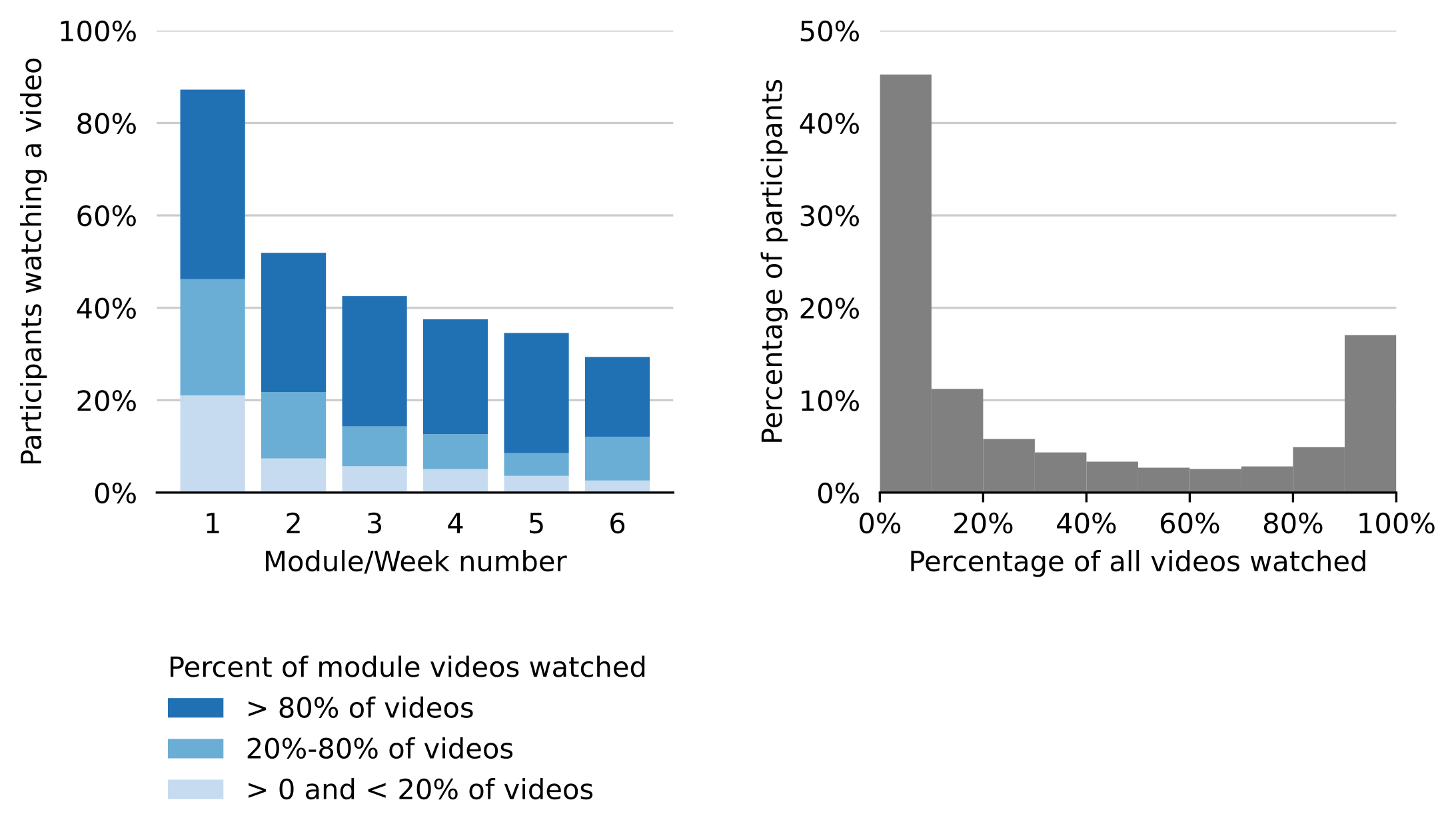


**S2 Fig. Video watching.** (A) Percentage of participants who watched more than one video who watched: less than 20%, between 20%-80%, and more than 80% of the module videos in each week. (B) Histogram of the percentage of all available videos watched by participants (e.g. 44% of those with any course activity watched between 0% and 10% of the module videos).

Once participants start watching the videos for a week/module, most of them go on to watch the majority of the week's videos. As S2 Fig shows, those who watch at least one video in a week usually watch more than 20% of the videos for that week/module, and over half watch more than 80% of the videos for the week/module. Overall, participants tend to either watch only a few course videos (55% watch less than 20% of the videos), or watch nearly all of the videos in the course (22% watch 80% or more of the videos).

### **Peer-graded assessments**

There are three PGAs during the course, during modules 3, 4, and 6. See the main text for a description of the PGAs.


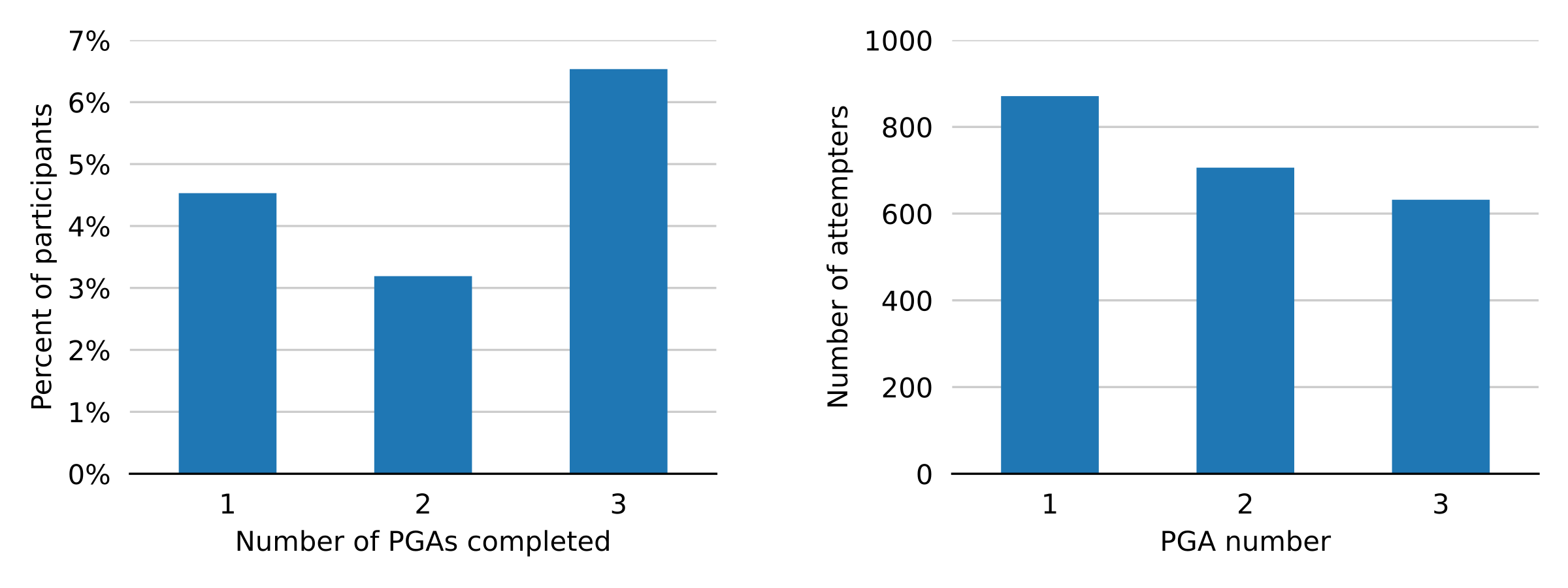


**S3 Fig. Peer-graded assessment completion.** (A) Histogram of PGA completion. Users who never engaged with any part of the course are excluded from the denominator. The fraction of participants who completed zero PGAs (86%) is not shown. (B) Number of participants who completed each PGA.

Participants who attempted a PGA were highly likely to also engage in other course activities such as taking quizzes or watching videos. 90% of PGA attempters completed four or more quizzes and 86% watched at least one video from 4 or more modules, compared with 15% and 25%, respectively, of non-PGA attempters. The dramatic difference in course engagement is strongly evident in S4 Fig, below. PGA attempters are 6 times more likely to have completed the course.


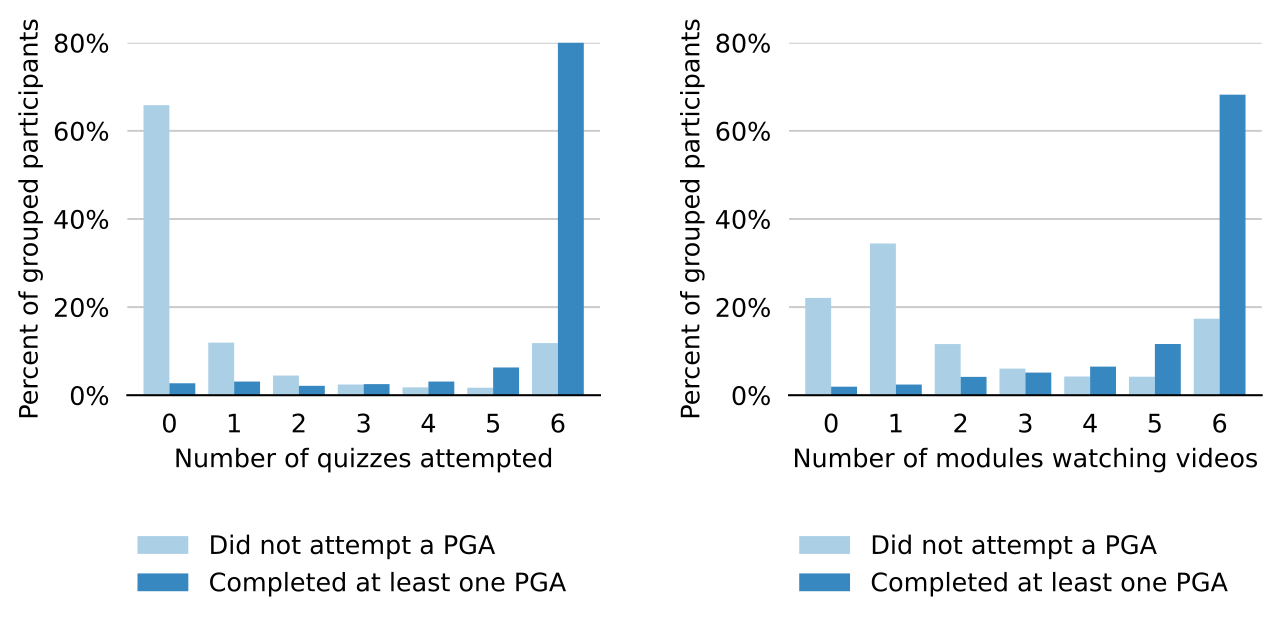


**S4 Fig. Correlation of PGA and quiz and video activity.** (A) Number of quizzes completed for PGA attempters and non-PGA attempters. (B) Number of weeks/modules watching > 1 video for PGA attempters and non-PGA attempters.

### **Learners**

The activity data above indicates three notable patterns:

- Activity dropped off significantly after the first, and to a lesser extent, the second course modules.
- For quiz completion and video watching, there is a bimodal distribution of participants.
- More participants watched videos than attempted quizzes or PGAs.
- Those who attempted a PGA were highly likely to engage in other course activities.

Given these patterns, it is appropriate to distinguish those participants who were significantly engaged with the course material from those who were not. We define learners as those participants who meet any of the following criteria:

- Watched at least one video from at least three different modules. This means the participant engaged with material from half of the course modules. And while the threshold is a single video, 77% of those who watch at least 1 video for a module watch at least 50% of the module’s videos.
- Attempted at least two quizzes. While quizzes are low-risk activities that do not demand much time, they do require participants to assess their understanding and learning. We observe a significant difference in the number of people who take one vs. two quizzes.
- Attempted at least one PGA. PGAs require a significant investment of time and thought by participants. 89% participants who do at least 1 PGA also meet one of the criteria above for quizzes or video watching, but the 11% who don’t have still significantly engaged with the course content in the process of doing the PGA.


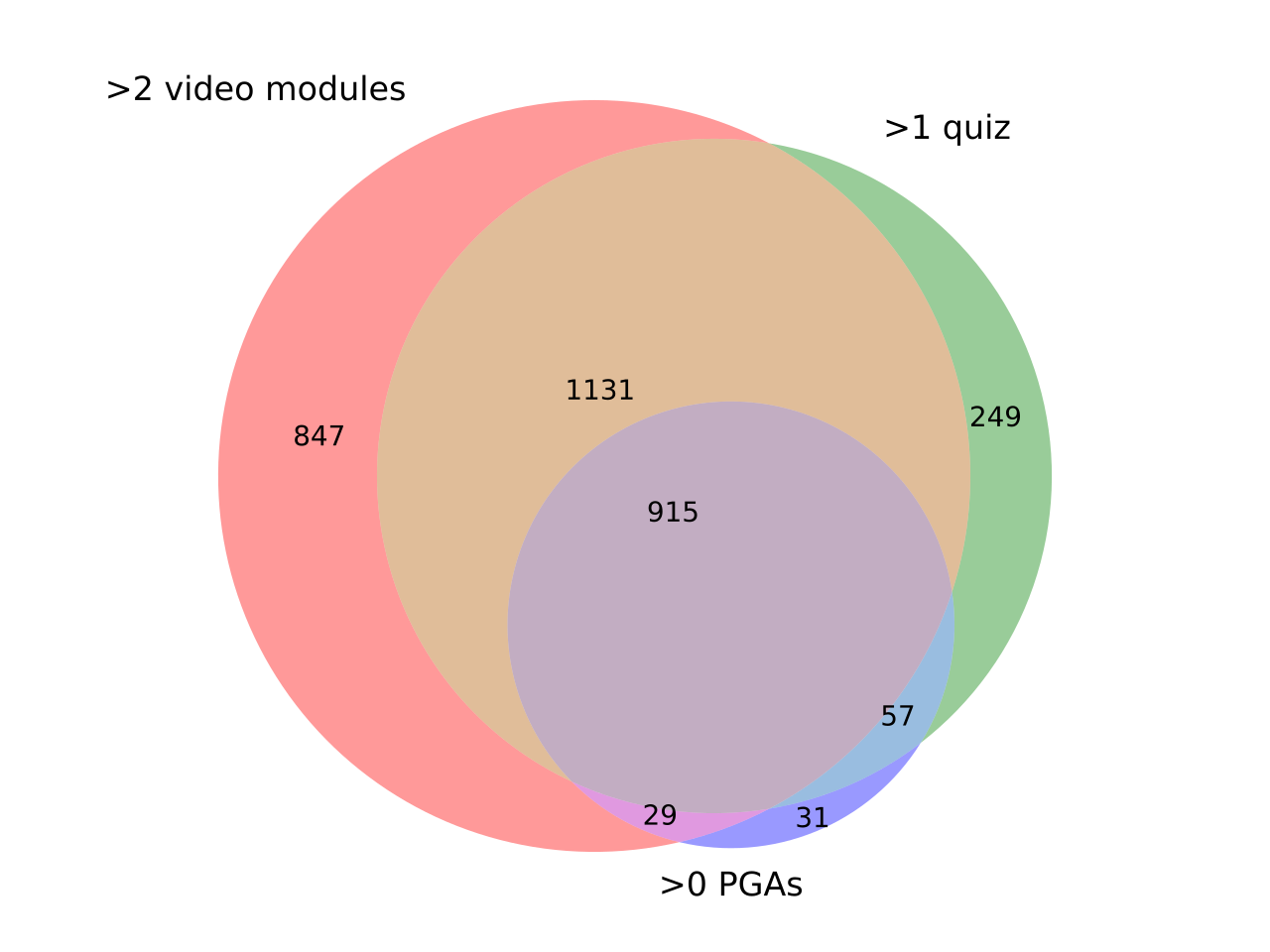


**S5 Fig.** **All learners: Venn diagram showing the extents of and overlap between different categories of Learners.** Three primary sets are participants who watched more than two modules’ videos in rose; attempted at least one PGA in purple; and attempted at least two quizzes in green. The other four colors represent the union of pairwise unions (teal, magenta, and beige) and the union of the three (dusty lilac).

In defining learners in this way, we grouped two primary groups:

- Auditors who primarily watch videos. 26% of learners only meet the definition of learner based on watching videos from 3 or more modules.
- Course completers who watch videos and attempt quizzes and/or PGAs, and meet the minimum requirements for course completion. 84% of people who attempt at least two quizzes or 1 PGA ultimately complete the course; this is 53% of total learners.

Auditors engaged in fewer modules on average than course completers (S5 Fig). They were more likely to only watch videos in three or four weeks than were course completers, and auditors were less likely to watch videos in all six weeks compared to course completers. Learners who did some quizzes or PGAs, but did not complete the course, engaged with videos in a pattern more similar to auditors than completers.


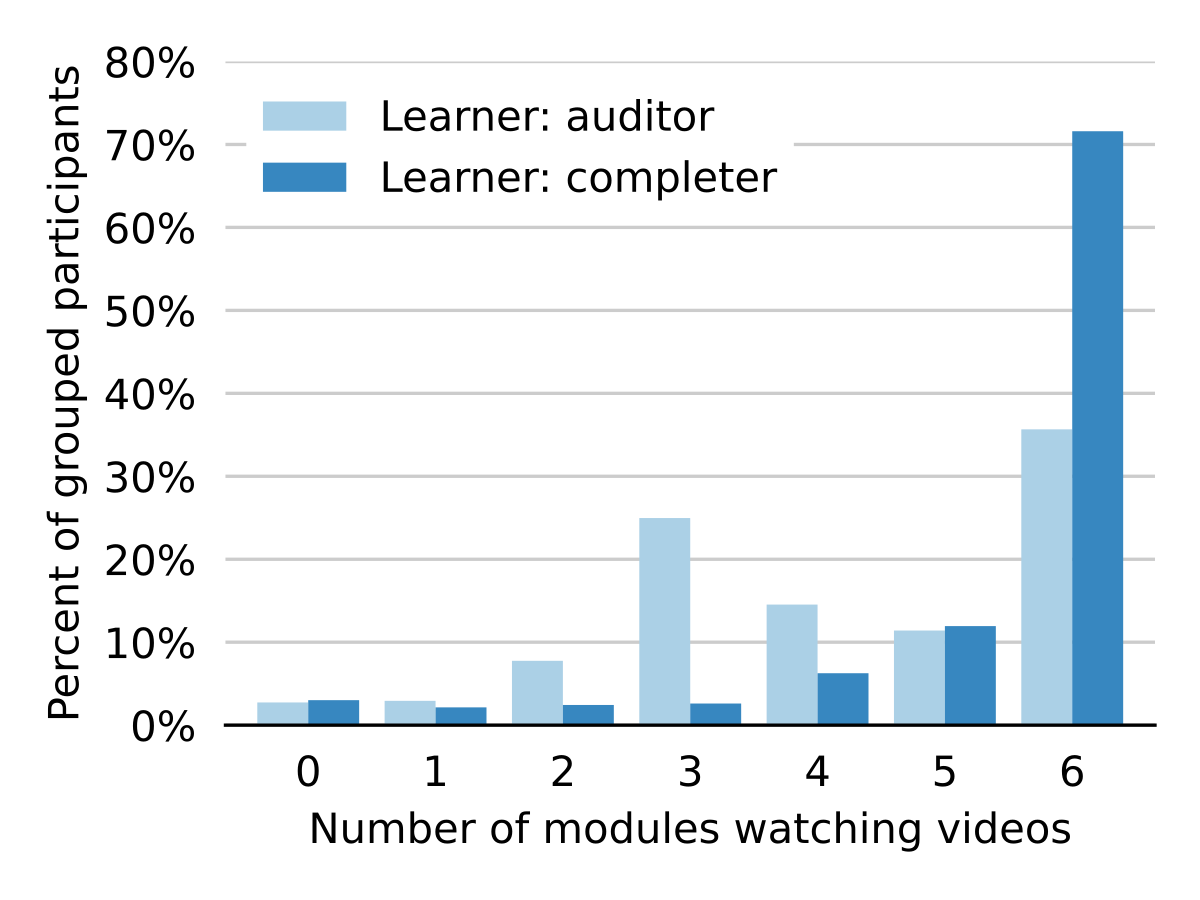


**S6 Fig. Number of weeks/modules watching > 1 video by learner category.**

### **Course completion**

Participants could complete the course by completing a combination of quizzes (weighted 60% together for four highest scores) and PGAs (individually weighted 10%, 10%, and 20%) to get an overall score of 50%. Some completers did more quizzes and PGAs than are required to meet the completion criteria. S4 Table shows the number of quizzes and PGAs done by those who completed the course. The most common way to complete the course, chosen by 40% of completers, was via six quizzes and no PGAs.

**S3 Table. Activity by course completers.**

|  |  | **Number of PGAs** | | | |
| --- | --- | --- | --- | --- | --- |
|  |  | **0** | **1** | **2** | **3** |
| **Number of Quizzes** | **0** | 0% | 0% | 0% | 0% |
|  | **1** | 0% | 0% | 0% | 0% |
|  | **2** | 0% | 0% | 0% | 0% |
|  | **3** | 0% | 0% | 0% | 0% |
|  | **4** | 4% | 1% | 0% | 0% |
|  | **5** | 5% | 1% | 1% | 1% |
|  | **6** | 40% | 10% | 11% | 25% |

S3 Table Legend: Aggregated activity across all instances of the introductory course.

## **Course activity of survey respondents**

For the first two instances of the introductory course, we can link survey responses with course activity. This allowed us to see what type of participants completed the two surveys. The majority of pre-course survey respondents were learners, and the majority of post-course survey respondents were not only learners, but also completers.


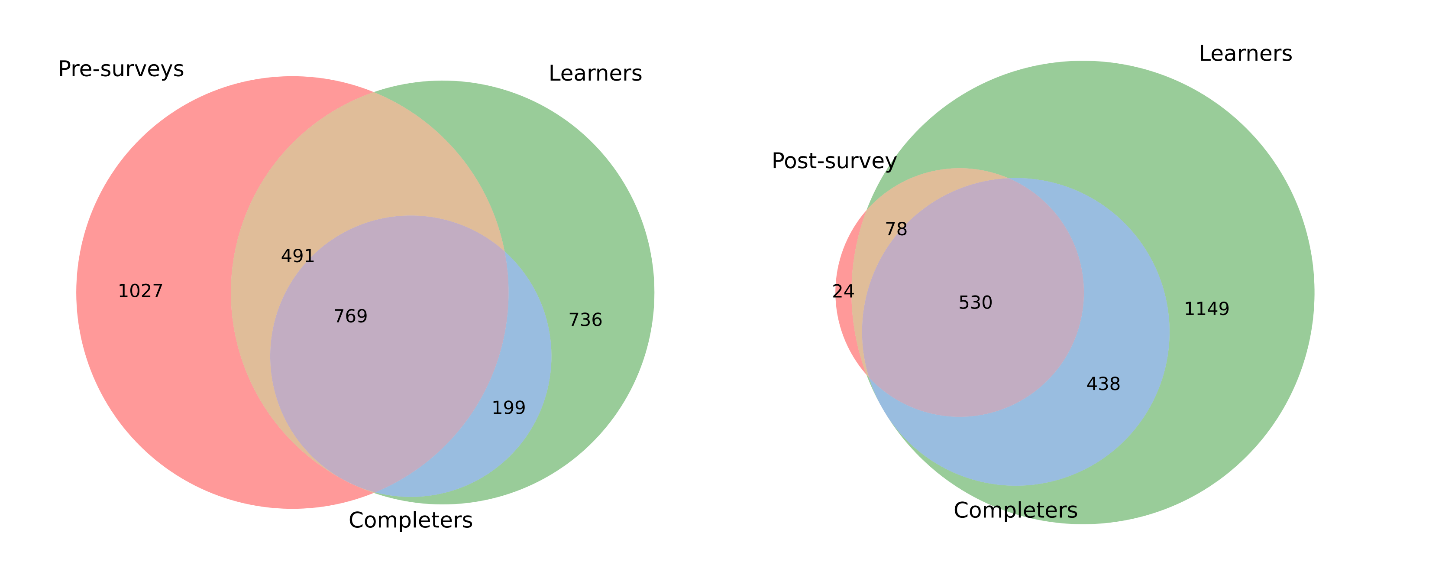


**S7 Fig. Overlap between survey respondents, learners, and completers for the first two instances of the introductory course.** (A) Pre-survey data and (B) Post-survey data demonstrating a high level of homopholy between survey respondents and learners and completers.

For the first two instances of the course, we can examine course activity by demographic characteristics reported on the surveys (S1 Table). Overall, the demographics of pre-survey respondent non-learners and learners were not drastically different. However, learners comprised a higher proportion of respondents from CIRTL institutions and respondents preparing to teach compared to non-learner respondents, and a slightly higher proportion of those who intended to participate in an MCLC when they took the pre-survey.

There were no significant differences between respondents' roles (faculty, post-doc, student) with respect to their likelihood to be engaged in the course as a learner, but among learners, postdocs were disproportionately more likely complete the course (22% of completers were postdocs, compared to 16% of auditors and 12% of other learners). Learners from CIRTL institutions and those intending to teach were also disproportionately more likely to complete the course.

**S4 Table. Pre-Course survey respondents' demographics by course engagement.**

|  | | **Non-learner** | **Learners (auditors)** | **Learners (completers)** | **Learners (other)** |
| --- | --- | --- | --- | --- | --- |
| **Count*** | | 1023 | 345 | 769 | 146 |
| **% of Pre-survey Respondents** | | 45% | 15% | 34% | 6% |
| **Institution** | |  |  |  |  |
|  | CIRTL member | 23% | 28% | 35% | 27% |
|  | Non-CIRTL | 77% | 72% | 65% | 73% |
| **Role** | |  |  |  |  |
|  | Doctoral student | 29% | 30% | 29% | 29% |
|  | Post-doctoral researcher | 18% | 16% | 22% | 12% |
|  | Faculty | 22% | 23% | 21% | 21% |
|  | Other | 30% | 31% | 28% | 38% |
| **Preparing to teach** | |  |  |  |  |
|  | True | 70% | 69% | 82% | 68% |
|  | False | 30% | 31% | 18% | 32% |
| **Plan to participate in MCLC** | |  |  |  |  |
|  | Yes | 29% | 31% | 30% | 37% |
|  | No | 25% | 27% | 28% | 23% |
|  | I'm not sure | 45% | 41% | 42% | 38% |

Table S4 Legend: The first two instances of the course only; only respondents taking the pre-course survey are included.

### **Motivations for taking the course**

Pre-course survey respondents were asked to rank the importance of different factors in their decision to enroll in the course. The most significant factor was “to enhance my STEM teaching skills,” with 93% of respondents ranking this as “Important” or “Very important.” The top three factors are all related to improving teaching and learning in the classroom. The least significant factors were “to earn credentials for my CV,” “to earn a statement of accomplishment,” and “curiosity about online courses.” Interestingly, the importance of motivating factors did not differ notably by role or by the respondent’s ultimate level of engagement with the course.

For most participants, peer interaction was less important than developing skills. Only 51% of survey respondents listed “connecting with other educators” as being “Important” or “Very important” in their decision to take the course.

**S5 Table. Motivations for Taking the Course.**

| **Enhance STEM teaching skills** | 93% |
| --- | --- |
| **Enhance learning of my students** | 89% |
| **Skills for my career** | 87% |
| **Developing a teaching plan** | 74% |
| **Connecting with other educators** | 49% |
| **Earn credentials for CV** | 49% |
| **Earning a statement of accomplishment** | 31% |
| **Curious about online courses** | 23% |

Table S5 Legend: Top 8 reasons stated as motivation for taking the course.

### Helpfulness of course components

Post-course survey respondents (84% of whom completed the course) ranked course videos as the most helpful learning components (S8 Fig), followed by PGAs and participation in an MCLC.


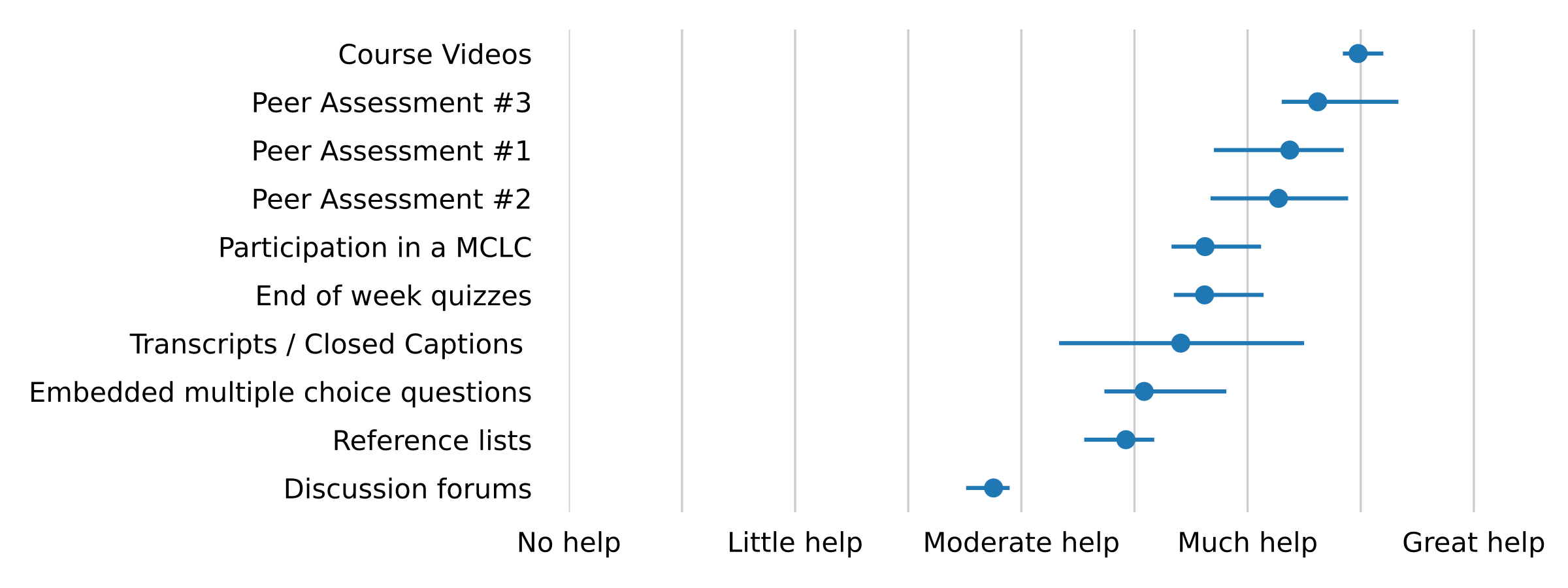


**S8 Fig. Post-Course Survey: Helpfulness of Course Components.** Respondents who did not answer the questions are excluded from the averages. Points show overall average across respondents. Error bars show the range of averages across course instances.

### **Learning indicators**

The post-course survey includes two series of questions that allude to learning gains in the course. The first is self-reported retrospective gains in interest in course topics and confidence applying skills covered in the course. Respondents reported the greatest gains in implementing and understanding the material from the course and wanting to discuss teaching with colleagues.


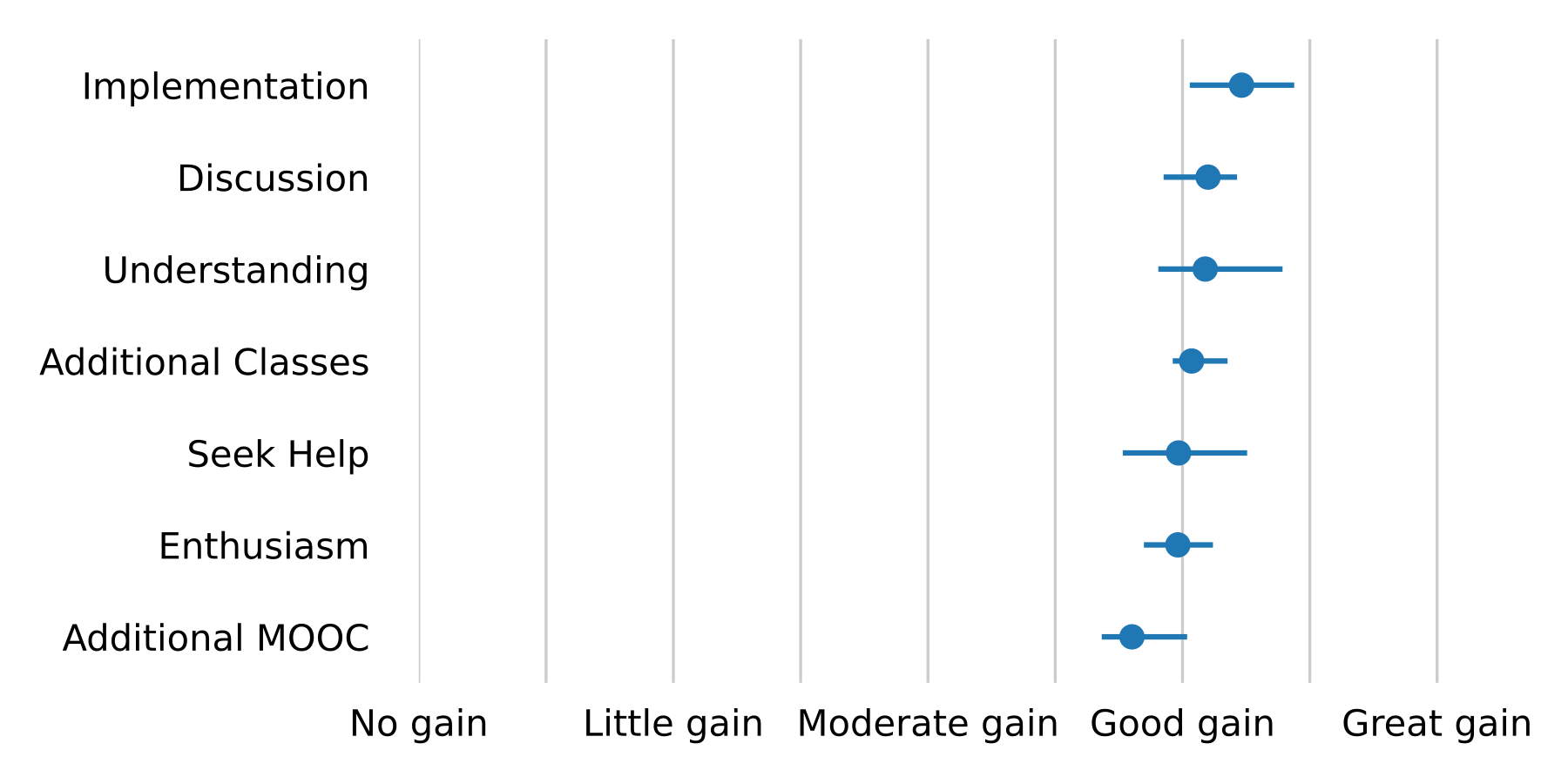


**S9 Fig. Self-Reported Learning Gains.** Respondents who did not answer the questions are excluded from the averages. Points show overall average across respondents. Error bars show the range of averages across course instances. Full question options are as follows:

- Enthusiasm: Enthusiasm for STEM teaching and learning
- Discussion: Interest in discussing teaching and learning with colleagues and friends
- Additional Classes: Interest in additional classes related to teaching and learning
- Additional MOOC: Interest in an additional MOOC related to teaching and learning
- Understanding: Confidence that you understand the material covered
- Implementation: Confidence that you can implement some of the teaching and learning strategies covered in class
- Seek Help: Willingness to seek help from faculty or peers regarding teaching and learning

The second series of questions that touches on learning outcomes concerns familiarity with course concepts, see S10 Fig. The same concepts are also included on the pre-course survey. However, since pre- and post-course survey respondents can only be linked for the first two instances of the course, analysis of individual gains can only be done for those instances. A figure showing average gains is included in the main paper.

Because the pre- and post-course surveys respondents were distinctly different groups, and most post-course survey respondents were a non-random subset of pre-course survey respondents, we cannot accurately compare pre- and post-course survey averages where individual response cannot be paired. For the other instances of the course, we can look at average familiarity ratings from the post course surveys.


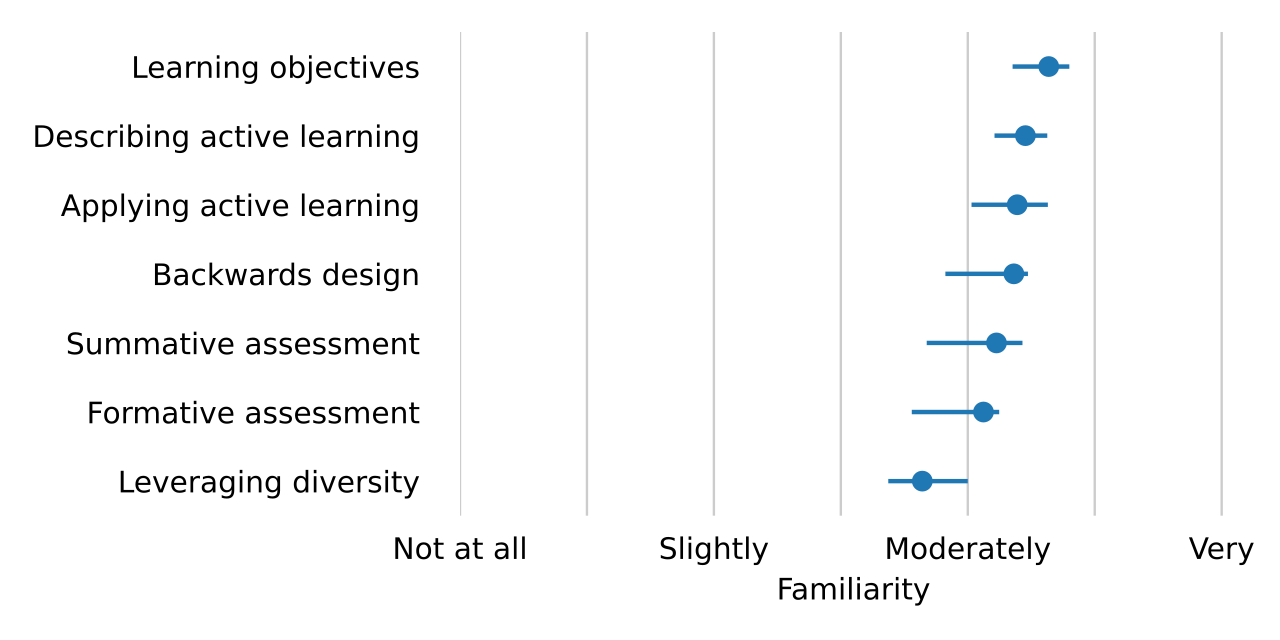


**S10 Fig. Post-course Familiarity with Course Concepts.** Respondents who did not answer the questions are excluded from the averages. Points show overall average across respondents. Error bars show the range of averages across course instances.

## **Learning community/MCLC engagement**

### **MCLC participation**

We do not have direct data on MCLC participation. The pre- and post-course surveys asked, respectively, about respondents' intent to participate in an MCLC and whether they did participate in an MCLC (S7 Table). Self-reported participation rates do not differ much based on participation intention, but those who did intend to participate in an MCLC were almost twice as likely as those who didn’t intend to participate to report participating on the post-course survey. Overall, 33% of post-course survey respondents report participating in an MCLC.

**S6 Table. MCLC Intent to Participate vs. Self-Reported Participation.**

|  | **Self-Reported Participation** | | |
| --- | --- | --- | --- |
|  | Yes | No | No response |
| **Intent** |  |  |  |
| Yes | 10% | 14% | 77% |
| I'm not sure | 7% | 15% | 78% |
| No | 6% | 17% | 77% |
| No response | 29% | 51% | 20% |
| All | 9% | 17% | 74% |

Table S6 Legend: Data is from the first two instances of the course and includes only the respondents who took both the pre- and/or post-course surveys. Intent is from the pre-course survey, while self-reported participation is from the post-course survey. No response indicates the individual either did not take the survey or did not answer the relevant question.

### **Motivation for MCLC participation**

Pre-course survey respondents who intended to participate in an MCLC were also asked about their motivations for doing so. S8 Table shows the proportion of pre-course survey respondents who intended to participate in an MCLC who reported each motivation. Social interaction was a primary motivator for those intending to participate in an MCLC.

**S7 Table. Pre-Course Survey Motivation for MCLC Participation.**

| **Motivation for MCLC Participation** | **Total** | **Percent** |
| --- | --- | --- |
| The opportunity to interact with peers | 579 | 54% |
| The opportunity to discuss course materials and assignments | 510 | 48% |
| The opportunity to meet others interested in teaching and learning | 501 | 47% |
| The opportunity to receive feedback on my teaching and learning practices | 450 | 42% |
| As a way of encouraging myself to keep up with the course material | 437 | 41% |
| The opportunity to interact with a facilitator | 383 | 36% |
| I do not plan to participate in a MCLC | 188 | 18% |
| I was required to participate in a local learning community for this MOOC as part of my academic program | 78 | 7% |
| Other reason for participating in a MCLC | 51 | 5% |
| I am not sure if I will participate in a MOOC-centered learning community | 11 | 1% |

S7 Table Legend: Top ten reasons stated as motivation for participation in a MOOC-centered learning community.

# **Data files**

Data files and analysis code are available at https://doi.org/10.5281/zenodo.7508428. These files include the data necessary to reproduce the analysis in the main paper and this supplementary file.

**References**

[1] Very limited and poor quality data on participants’ age, educational attainment, gender and country are the only demographic variables available from these platforms. We did not find this data to be reliable enough to include in the analysis.

Supporting Information Captions

**S1 Table. Enrollment and demographics from 2014-2018 in An Introduction to Evidence-based Undergraduate STEM Learning**. Enrollment data, rate of pre-survey response, demographics of participants, location in a CIRTL institution, status or role, teaching preparation as motivation, intention to participate in an MCLC (pre-survey) and academic discipline.

**S2 Table. Post-course respondent demographics from 2014-2018 in An Introduction to Evidence-based Undergraduate STEM Learning.** Enrollment data, rate of post-survey response, demographics of participants, location in a CIRTL institution, status or role, actual participation in an MCLC (post-survey) and academic discipline.

**S1 Fig. Quiz completion.** (A) Histogram of total number of quizzes completed. (B) Number of participants who completed each quiz.

**S2 Fig. Video watching.** (A) Percentage of participants who watched more than one video who watched: less than 20%, between 20%-80%, and more than 80% of the module videos in each week. (B) Histogram of the percentage of all available videos watched by participants (e.g. 44% of those with any course activity watched between 0% and 10% of the module videos).

**S3 Fig. Peer-graded assessment completion.** (A) Histogram of PGA completion. Users who never engaged with any part of the course are excluded from the denominator. The fraction of participants who completed zero PGAs (86%) is not shown. (B) Number of participants who completed each PGA.

**S4 Fig. Correlation of PGA and quiz and video activity.** (A) Number of quizzes completed for PGA attempters and non-PGA attempters. (B) Number of weeks/modules watching > 1 video for PGA attempters and non-PGA attempters.

**S5 Fig. All learners: Venn diagram showing the extents of and overlap between different categories of Learners.** Three primary sets are participants who watched more than two modules’ videos in rose; attempted at least one PGA in purple; and attempted at least two quizzes in green. The other four colors represent the union of pairwise unions (teal, magenta, and beige) and the union of the three (dusty lilac).

**S6 Fig. Number of weeks/modules watching > 1 video by learner category**.

**S3 Table. Activity by course completers.** Aggregated activity across all instances of the introductory course.

**S7 Fig. Overlap between survey respondents, learners, and completers for the first two instances of the introductory course**. (A) Pre-survey data and (B) Post-survey data demonstrating a high level of homophily between survey respondents and learners and completers.

**S4 Table. Pre-Course survey respondents' demographics by course engagement.** The first two instances of the course only; only respondents taking the pre-course survey are included.

**S5 Table. Motivations for Taking the Course.** Top 8 reasons stated as motivation for taking the course.

**S8 Fig. Post-Course Survey: Helpfulness of Course Components.** Respondents who did not answer the questions are excluded from the averages. Points show overall average across respondents. Error bars show the range of averages across course instances.

**S9 Fig. Self-Reported Learning Gains Respondents who did not answer the questions are excluded from the averages**. Points show overall average across respondents. Error bars show the range of averages across course instances. Full question options are as follows:

- Enthusiasm: Enthusiasm for STEM teaching and learning
- Discussion: Interest in discussing teaching and learning with colleagues and friends
- Additional Classes: Interest in additional classes related to teaching and learning
- Additional MOOC: Interest in an additional MOOC related to teaching and learning
- Understanding: Confidence that you understand the material covered
- Implementation: Confidence that you can implement some of the teaching and learning strategies covered in class
- Seek Help: Willingness to seek help from faculty or peers regarding teaching and learning

**S10 Fig. Post-course Familiarity with Course Concepts.** Respondents who did not answer the questions are excluded from the averages. Points show overall average across respondents. Error bars show the range of averages across course instances.

**S6 Table. MCLC Intent to Participate vs. Self-Reported Participation.** Data is from the first two instances of the course and includes only the respondents who took both the pre- and/or post-course surveys. Intent is from the pre-course survey, while self-reported participation is from the post-course survey. No response indicates the individual either did not take the survey or did not answer the relevant question.

**S7 Table. Pre-Course Survey Motivation for MCLC Participation.** Top ten reasons stated as motivation for participation in a MOOC-centered learning community
