# Supplementary material for: Preparing future STEM faculty through flexible teaching professional development
Source: PLoS One. 2023 Oct 12;18(10):e0276349. doi: 10.1371/journal.pone.0276349 (PMC10569627; doi:10.1371/journal.pone.0276349)
Supplement: Attachment — Submitted filename: Response to reviewers.docx [file pone.0276349.s002.docx]

Response to reviewers

PONE-D-22-27145

Title: Preparing future STEM faculty through flexible teaching professional development

Dear editor and reviewers,

We very much appreciate your close reading of the manuscript and we took very much to heart your questions and suggestions for revisions. As you can see by the responses below, we agreed with you, and made changes to the manuscript which we believe, and hope you will too, provide a significant improvement in clarity, connectivity, and readability.

This response is organized in the following way: Each of the major changes (small wording or grammatical changes have not been highlighted) is listed in tabular format together with the page number from the tracked-changes resubmitted manuscript. These are in order of appearance in the manuscript. Following the table, the editorial and review suggestions are listed together with the numbered change that reflects that particular question(s).

List of changes per comments from the review:

| Change # | Page # | Change | Notes |
| --- | --- | --- | --- |
| 1 | 1 | Removed ‘nationwide’ from title to respond to the international reach of Plos One | Response to referee #1 |
| 2 | 2, 9 & 11 | Changed the percentage of completers to the average over all instances of the course rather than as an average of course completion rates. This eliminates confusion over the relatively minor and not meaningful difference between an overall average and the average of course rates of completion. | Response to referee #2 |
| 3 | 3 | Modified the description of marginalized students and the positive impact of inclusive teaching in reducing outcome disparities | Response to referee #2 |
| 4 | 5 | Added central guiding questions. Additionally, these now organize the sections on results to help guide the reader. | Response to referee #1 |
| 5 | 6, 7 & 8 | Modified the Methods section in the following ways: Under a new subsection ‘Course logistics,’ added a clear statement of what defines a ‘completer;’ and provided a detailed description of the IRB review, exempt status as program evaluation, and anonymous and optional survey methodology. Under a new subsection ‘Data’, shifted detailed description of the data sources from the supplemental document to the methods, and added two references to full MCLC evaluation reports on p.8. A section on ‘analysis,’ includes the types of analyses completed and added references for the analysis tools. | Response to editors requesting ethics and IRB process in manuscript; and response to referees #1 and #2 |
| 6 | 9, 10, 11 | Modified the Results section: Created a new subsection ‘Learner engagement and target audience,’ that clearly describes the differences of ‘learner’ and ‘auditor’ etc. A new consort diagram as figure 2 has been added as well, to help clearly define each term and its relation to others. Next, we removed the table since it was confusing and the new text and figure hopefully provides a clearer picture; also a new table 1 has been added to the supplemental materials. Country of origin data is added. Further, data on participant motivation is brought from supplemental materials into the manuscript to support the conclusions of the guiding questions. | Response to referees #1 and #2 |
| 7 | 14 &15 | Details of the demographics of participants have been brought forward from the supplement. Also, specific elements of the supplement are indicated where they support the manuscript text. | Response to referees #1 and #2 |
| 8 | 15 | The statistical test used to determine significance are now included. | Response to referee #2 |
| 9 | 16 | The results section entitled, subsection “Learning and Confidence to Apply Content” now follows the guiding questions, adding clarity and organization. | Response to referees #1 and #2 |
| 10 | 18 & 19 | The section on Open Education Resources has been modified to clearly state where the data is obtained, and how individuals access the content. | Response to referee #2 |
| 11 | 21 | The discussion section has revised the section on the CIRTL Network contribution to the project. The discussion section now includes illustration of how other projects have leveraged and utilized our approach for professional development of graduate students and postdocs. | Response to referee #2 |
| 12 | 18 | A paragraph on study limitations and recommendations for future work has been added to the end of the results section just prior to discussion. | Response to referee #2 |
| 13 | 29 | The image of prevalent words (word cloud or word diagram) has been deleted, as has the final single sentence. These were never intended for this article. | Response to referee #2 |
| 14 | multiple | The references were re-ordered and formatting corrected. | Response to editors |
| 15 | multiple | All headings, sub-headings, figure & table titles, captions and legends in the manuscript and supporting materials have been reformatted to PLOS ONE style requirements. | Response to editors |
| 16 | 29-32 | All supporting material figure and table titles, captions and legends added to the end of the manuscript. | Response to editors |
| 17 | Supporting materials | The supporting materials was shortened, with several sections removed that were not referenced in the main manuscript. All the figures and tables were properly formatted. | Response to referee #2; response to editors |

**Journal requirements:**

1. Please ensure that your manuscript meets PLOS ONE's style requirements.

We have reformatted and followed style guidelines throughout. See changes #s 14, 15 & 16 in the changes table.

2. Please provide additional details regarding participant consent.

We have provided additional details in the manuscript under “Methods” regarding participant consent. This also replaces the ethics statement in the submission upload.

3. We note that you have stated that you will provide repository information for your data at acceptance. Should your manuscript be accepted for publication, we will hold it until you provide the relevant accession numbers or DOIs necessary to access your data. If you wish to make changes to your Data Availability statement, please describe these changes in your cover letter and we will update your Data Availability statement to reflect the information you provide.

A DOI is provided in the supporting materials: Data files and analysis code are available at https://doi.org/10.5281/zenodo.7508428. These files include the data necessary to reproduce the analysis in the main paper and this supplementary file. Please make this change in the Data Availability statement.

4. Please include your full ethics statement in the ‘Methods’ section of your manuscript file. In your statement, please include the full name of the IRB or ethics committee who approved or waived your study, as well as whether or not you obtained informed written or verbal consent. If consent was waived for your study, please include this information in your statement as well.

Our revised ethics statement is identical to what is now in the Methods section: We invited course participants to take surveys for project evaluation. These surveys and learning activity data obtained from the course platforms were determined “not human subjects research” by the Michigan State University Institutional Review Board (IRB), hence formal consent was not required. Interviews of MCLC facilitators were determined to be Exempt by the IRB at University of Colorado Boulder (protocol 15-0658). All interview respondents provided informed consent in writing.

5. Please include captions for your Supporting Information files at the end of your manuscript, and update any in-text citations to match accordingly.

Captions have been added to the end of the manuscript and in-text modifications as well. See change #16 in the changes table above.

**Response to reviewer #1:**

We very much appreciate the careful reading of our manuscript and recommendations of reviewer #1. We agree and have made the suggested changes.

1. In the title, the term "nationwide" is not specific to which nation so that should be added as this is an international journal.

“Nationwide” was removed from the title. See change #1 in the changes table above.

2. Perhaps I missed it, but were there any "research questions" presented and answered? At times the results felt overwhelming and lacking direction as I wasn't sure what knowledge gap they were filling. Please specify what some of the questions were prior to the start of the initiative and if/how they were answered.

Guiding questions have been added. See change #4 in the changes table above. These also now provide greater clarity by organizing the results section.

3. I was troubled by the use of the label "learners" for the "participants who complete at least two quizzes, complete a PGA, or watch videos from at least three of the six course modules". The label learners implied that they learned something, but really they "participated", so why not call them participants? I think that label is misleading somewhat.

We have rewritten the description of the terms of participant engagement throughout the manuscript and supporting materials to reflect this comment and a similar comment from reviewer #2. We have added a new fig 2 which also distinguishes between the different types of engagement. We further qualify the term ‘learner’ to indicate completion of assessments and course activity, acknowledging that we are not measuring direct work product. See changes #5 & #6 in the changes able above.

4. In the discussion on line 278 the authors stated, "Our target audience of STEM graduate students and postdocs"... perhaps I missed it, but was this identified as the target audience earlier in the paper? Were the demographic or job characteristics of the participants in this study described? That would be helpful information.

We have brought forward demographic information from the supporting materials into the main manuscript to clearly identify our target audience. We have included appropriate additional references to the fuller tables in the supporting materials. See change #7 in the changes table above.

**Response to reviewer #2:**

We thank reviewer #2 for careful reading of and insightful comments about our manuscript. We agree with nearly all the suggestions, and believe that the revisions in response to recommendations have significantly improved the manuscript.

1. In this study, the authors are overall trying to demonstrate the effectiveness of the model of using MCLCs to accompany a MOOC in improving and sustaining learner engagement. The participation tied to survey data seems to make this case but paper is challenging to read and follow at times, which may be remedied in part with some substantial revisions to Methods section, clarification of terms and definitions used throughout the Results, as well as a more thoughtful and focused effort to reference supplementary materials relevant to the study.

The Methods section has been substantially revised to clarify terms and definitions; guiding questions have been added which helps organize the Results; and supporting materials have been more thoughtfully and specifically referenced in the manuscript. See changes #s 4, 5 & 6 in the changes table above.

There also seemed to be reliance on membership in the CIRTL network for effective implementation of MCLCs. The authors may want to consider how aspects of the model itself can be adopted more broadly in the absence of the CIRTL network.

The discussion now includes a section on the reliance on CIRTL Network and opportunities for other networks; see change #11 in the changes table above.

2. Line 45-46: This reviewer suggests that the authors consider moving away from a deficit-based description of the equity and opportunity gaps we see in higher education with reference to student “performance”, swapping this term instead for “outcomes”. Also suggest authors avoid labeling communities of color as “minorities” as it implies status as opposed to population percentage. Suggest rephrasing this and other sentences throughout manuscript. For example, “… reduce the disparities in outcomes between marginalized students who are historically underrepresented in STEM and their [white] peers”. Or “… reduce the disparities in outcomes between STEM students in racially minoritized groups and those in the dominant group”. Please see this article published by the National Association of Hispanic Journalists for context and other ideas for alternative phrasing ideas when referring statistics and data describing communities of color.

The language has been modified as recommended. See change #3 in the changes table above.

3. Line 97: Materials and Methods. The authors did a nice job describing the structure of the MOOC and MCLCs. What’s missing, however, are the methodological and analytical details of the actual research study (some are buried in the Results but not in a consistent way). In other words, they need to provide a description of the methods by which they conducted their research on the MOOC and MCLC participants (data collection and analysis). What was the protocol for administering the pre- and post-surveys? Can the authors provide the IRB-approved survey instrument in the supplementary materials? How were the data analyzed? What statistical tests were undergone to examine the validity of their results? What was the protocol for interviewing MCLC facilitators? Were they asked specific questions? There also seemed to be some informal assessment done of OER utility from colleagues (line 266-267) as well as analytics pulled from the MOOC website itself (line 268). None of this methodological detail is provided thus making the Results, overall, difficult to follow or evaluate.

The Methods section was revised to include a specific subsections on the IRB for the courses and MCLCs; a subsection on data describing exactly where the data came from; a subsection on the analysis approach; and where statistical results are presented, the specific test applied. See changes #s 5, 6, 8, & 9 in the changes table above.

Regarding the surveys and interview protocols, the supporting materials detail the vast majority of the responses in tables and data and figures presented. We have also added detail on the interviews in the manuscript text and linked to the full MCLC evaluation reports, references #29 & #30 on page 8 of the manuscript under the new subsection ‘Data.’ See change #8 in changes table above.

The OER section was revised to include specifics on the informal assessment. See change #10 in changes table above.

4. Line 110: Can the authors explain how they “encouraged” participation in the MCLC? Some additional details on how this was operationalized would be helpful.

Detail at the end of the Logistics subsection of the Methods section describes how participation as a potential MCLC facilitator and participant were/are marketed.

5. Line 116: Curious as to why access to the MCLC Facilitator Guides requires sign-up to be a facilitator. Are there communications or training that occurs with facilitators that necessitate limiting access to this resource? On the website (https://www.stemteachingcourse.org/learning-communities/mclc-facilitators), the Q/A video for facilitators states, “Video unavailable. This video is private.”

We are in the process of removing the video, as it was out of date. There is a lot of information provided in written form on the website. The reason we ask for email is to allow us to reach out to facilitators with any changes that come along in the course, so they can alert their local learning communities. For example, we have recently revised the module on inclusive teaching.

The CIRTL MOOC project did not have funds for facilitation training, but did do workshops at CIRTL and at other national meetings about the course and MCLCs. It is worth noting that in subsequent projects, The Postdoc Academy and the Inclusive STEM Teaching Project that some of us are also involved in, we have built in facilitator trainings as well as check-ins.

6. Line 133-134: What was the distribution of participants from the 60 different countries? Were the majority in the US and Canada given the institutional composition of the CIRTL Network? Is this an important demographic to point out with respect to the findings reported in this paper?

Average country of origin data is added to the results section describing demographic distributions. See change #6 in the changes table above. Further discussion was not engaged as there isn’t significant meaning. CIRTL distribution information is provided in the supporting materials in S1 Table and S2 Table.

7. Table 1: Mean % of learning completing the course and auditing the course do not add up to 100%. How did the authors characterize the remaining 4% in intro course and 21% in advanced course? Are these non-completers but still Learners based on definitions provided? Perhaps it would be helpful to define a non-completer in the table legend. According to Figure 1b, maybe these are the “Disengaged” and/or “Learner-other”? Or are the “Disengaged” the “non-learners (Line 171). It’s not clear. These terms need to be defined and used consistently as the subsequent sections of the paper refer to these terms when discussing survey results.

We have removed all data concerning the average of different course instance outcomes, as they are not germain or meaningful. This was the source of the confusion on Table 1. The data now describes the average over the instances of the course(s).

‘Learners,’ ‘non-learners,’ ‘auditors,’ and ‘completers’ are now fully and consistently described throughout the manuscript and supporting materials’ text, tables and figures. These two changes mean that percentages add to 100% and that the meaningless small group who were previously termed ‘other-learner’ are now included in the clarified distinctions.

See change #s 2 & 6 in the change table above.

8. Line 150: Reference to “course completion” is not consistent with descriptions in Table 1 legend.

Table 1 had averages of outcomes across course instances which are now removed. Table 1 is replaced by a simple consort diagram figure 2. The text has been clarified as well. See change #s 2 & 6 in the change table above.

9. Line 149: The amount of information and data analysis provided in the supplementary materials is substantial. And these three lines (147-149) in the primary manuscript do not really do the material justice. If this information is critical to the findings shared in the primary manuscript, this reviewer would like to see it summarized with reference to relevant supplementary tables and figures. One might argue that the participation, activity, and outcome data could be a separate paper and subject to its own independent review. It would be helpful if the authors could focus on what information from the supplementary materials is most critical to the findings of the primary manuscript.

The supporting information has been thinned, as the main manuscript refers many more times to the supporting information directly. Supporting table and figure captions have been added to the main manuscript as requested by the editor.

We considered the recommendation to create a separate paper on participation, activity and outcome data and concluded that (1) based on recommendations, we brought forward key aspects in the supporting materials that support our central guiding questions; and that (2) there was insufficient new and meaningful results to support an independent publication. See change #6 in the change table above.

10. Line 148: In referencing citation #30, please check formatting. Is this supposed to be a footnote?

Prior reference [30] is a paper. All references have been reformatted properly in Vancouver style except the few that are footnotes. See change #13 in the change table above.

11. Lines 161-167: Figure 1 panel (b) – define “Disengaged”, “Learner – other”, “Learner – auditor” and “Learner-completer”. These terms are only partially consistent with Table 1 or what is referenced in the text (Lines 155-157, 171-172). This lack of consistency is making it difficult to follow the text in reference to the figures and table.

‘Learners,’ ‘non-learners,’ ‘auditors,’ and ‘completers’ are now fully and consistently described throughout the manuscript and supporting materials’ text, tables and figures. See change #2 and #6 in the change table above.

12. Line 213: Since authors have a figure with two panels (a and b), the text should refer to each panel separately and discuss the significance of these differences, not just the apparent differences.

Text on p16 has been modified to refer to both panels.

13. Line 236: In referencing citation #35, please check formatting. Was this also intended to be a footnote?

Prior reference [35] has been properly formatted. See change #13 in the change table above.

14. Line 272: The Discussion provides a nice summary with some of the details that were missing in earlier parts of the paper. Suggest moving some of this information such as operationalization of MCLC recruitment (Line 110). Would like to see the author address some of the limitations of the study in this section as there were, for instance, issues with IRB approval linking demographics with survey data. This impacted the sample size and subsequent analysis.

The operationalization of MCLC recruitment is in the same paragraph under course logistics as the MCLC description early in the manuscript.

We have added a paragraph titled, ‘Limitations and recommendations for future work,’ at the end of Results just prior to the Discussion. See change #12 in the changes table above.

15. Additional comments: There was a wordle on page 27 and several supplementary figures (S1-S9). No reference to this information was provided in the primary text; the supplementary figures are discussed in the supplementary file referenced in line 149 of the primary text. The volume of information provided in the supplement is excessive. This reviewer recommends that the authors curate this information and include only that which is relevant to the findings of the primary manuscript with specific and thoughtful reference to supplementary figures and tables in the primary text. Such efforts would help to create a more focused read of the research study and appreciation of the findings from readers.

The supporting information has been thinned, as the main manuscript refers many more times to the supporting information directly. The word cloud has been removed. Supporting table and figure captions have been added to the main manuscript as requested by the editor. See changes #s 13 & 17 in the changes table above.
