# Supplementary material for: Preparing future STEM faculty through flexible teaching professional development
Source: PLoS One. 2023 Oct 12;18(10):e0276349. doi: 10.1371/journal.pone.0276349 (PMC10569627; doi:10.1371/journal.pone.0276349)
Supplement: Attachment — Submitted filename: Rev 2 Final Response to reviewer April 2023.docx [file pone.0276349.s003.docx]

Response to reviewer	April 2023

PONE-D-22-27145

Title: Preparing future STEM faculty through flexible teaching professional development

Dear editor and reviewer,

We very much appreciate your close reading of the manuscript and we took very much to heart your questions and suggestions for revisions. As you can see by the responses below, we agreed with you, and made changes to the manuscript which we believe, and hope you will too, provide a significant improvement in clarity, connectivity, and readability.

This response is organized in the following way: Each of the major changes (small wording or grammatical changes have not been highlighted) is listed in tabular format together with the page number from the tracked-changes resubmitted manuscript. These are in order of appearance in the manuscript.

| **Change #** | **Page #** | **Change** |
| --- | --- | --- |
| 1 | 2 | As advised, the abstract was revised to remove background, add additional results, and 1-2 sentences to connect to real-world applications of the outcomes of our study. |
| 2 | 5 | A sentence has been added to the guiding questions to identify for the reader the distinction between a hypothesis-driven versus an educational project with evaluation results. |
| 3 | 6 | Two sentences at the start of the ‘Methods’ section clarify for the reader the following sections and their connection to each of the guiding questions. |
| 4 | 8-9 | As advised, the Methods have been revised to identify which data source is associated with which guiding question and combined with the analysis section to clarify how the analysis was applied to answer the guiding questions. |
| 5 | 12 | As advised, the data displayed as (##/##%) has been revised and clarified. The number format refers to pre- and post-survey respondents, which has now been highlighted in the text. |
| 6 | 13 | In the Results, edited the start of the section answering guiding question three to clarify the connection between results and answering the guiding question. |
| 7 | 19 | As advised, limitations and implication for future work was moved to follow the discussion |
| 8 | 20 | As advised, one short paragraph taken from the discussion is repurposed as a conclusion. |
| 9 | 12 | As advised, removed some extraneous text from the Results |

**Comments by reviewer with itemized changes:**

Reviewer #3: Thank you for submitting your revised manuscript for reconsideration. It is apparent that a lot of hard work has gone into this work. The previous reviewer had indicated that the objectives were unclear and you had added these in the introduction. However, upon reviewing your paper the objectives remain unclear and particularly how your data addresses these questions remains unclear. I re-read the abstract after reviewing your manuscript to better understand your objectives and how these were met - but was unable to find a focus even after re-reading the abstract. Consider what is the hypothesis being tested in your paper and how this was proven/disproven by your data. Additionally, the manuscript is long and may benefit from being shortened to be succinct and focus on the primary question(s) being asked/addressed.

*The following revisions were made:*

1. *Revised abstract. Change #1.*
2. *Explanation in the text that this is not a hypothesis-driven study but rather a program evaluation with emergent themes and conclusions based upon findings. Change #2.*
3. *Revise how the objectives are connected to the data. Changes #3 and #4.*
4. *Removed extraneous results to the supplement. Change #9.*

My constructive / focused suggestions to address my above comments are as follows:

1. I would firstly recommend that the authors re-write the abstract. The abstract has too many words dedicated to background. Background is generally permitted to be 1 sentence. Methods should be briefly stated - looks like there is some attempt to do this. No results are presented in the abstract and this is the primary purpose of the abstract. Please revise the abstract and provide the pertinent results which address the objectives that you are trying to address. The abstract should end with 1-2 sentences addressing how the findings address the objectives and/or their real world applicability. The abstract should be able to stand alone and be informative to the reader providing pertinent information.

1. *Edited the abstract to cut the background and provide more results, and extend the outcomes to identify the real-world applicability. Change #1.*

2. The methods section does clearly not state how the data will be analyzed to answer the questions posed by the authors as their objectives. This should be revised accordingly.

1. *Edited the methods section to more clearly state this. Changes #3 and #4.*

3. Consider revising your results so that the subsections are designed to address each of the hypotheses/objectives being tested. This may provide the reader with better clarity to see the data addressing each of the questions you are testing.

1. *Reorganized the results in a more straightforward way, referring directly to each of the objectives. Change #6.*

4. Depending on the above, I would suspect that the results will need to be revised around the above structure.

1. *See above.*

5. It is traditional to place the limitations section after the discussion, and to add a conclusions section to the manuscript. The conclusion section would be a short paragraph to summarize succinctly how your manuscript addressed the questions/objectives/hypotheses and the real world appliability of the data you are presenting

1. *Moved the limitations section to after the discussion. Change #7.*
2. *Edited the conclusion - repurposed the last paragraph of discussion. Change #8.*

6. Consider removing any data and text that do not directly address the objectives/hypotheses. While I acknowledge that a lot of work has been done in this project by your team, for the reader it is very important that the manuscript clearly defines objects and whether these were met or not. Some of this additional text may be moved to the discussion where pertinent.

1. *Removed extraneous sections of the results. Change #9.*

Minor comments:

A. Consider not using abbreviations as key words. Abbreviations are not generally referenced by academic search databases. - I would ask that the editor/editorial staff overrule this comment if abbreviations as keywords are acceptable for use as keywords for PLOS ONE

1. *Asking the editor if “STEM” and MOOC” are allowed as a keywords.*

B. The authors present data with "Absolute number/percentage". This style is not denoted in the methodology. The more usual way of presenting this is "absolute number (%)". Consider revising for clarity as when reading the data it is unclear if you are reporting 2 different percentages, vs reporting a fraction, vs reporting absolute number/percentage as I believe is your intention.

1. *Revised the section that has the (number/number % sign) which actually refers to the same percentage, just one is pre- and one post-course survey. Change #5.*
