# Supplementary material for: Preparing future STEM faculty through flexible teaching professional development
Source: PLoS One. 2023 Oct 12;18(10):e0276349. doi: 10.1371/journal.pone.0276349 (PMC10569627; doi:10.1371/journal.pone.0276349)
Supplement: Attachment — Submitted filename: Rev 3 Response to review May_June 2023.docx [file pone.0276349.s004.docx]

Response to reviewers	June 2023

PONE-D-22-27145

Title: Preparing future STEM faculty through flexible teaching professional development

Dear editor and reviewers,

We are pleased that Reviewer #1 said, “Excellent work on revisions, this manuscript is ready for publication.” We appreciate the comment and the confirmation that Reviewer #1 feels we met the expectations with the prior revisions.

We are pleased as well that Reviewer #3 saw significant improvement. We very much appreciate the close reading of the manuscript and we took very much to heart the remaining suggestions for improvement and publication. As you can see below, we made changes to the manuscript based on reviewer comments which we believe, and hope you will too, provide a significant improvement in clarity, connectivity, and readability.

This response is organized in the following way: Each of the major changes is listed in tabular format together with the page number from the tracked-changes resubmitted manuscript. These are in order of comment by Reviewer 3 and close to their order of appearance in the manuscript.

| **Change #** | **Page #** | **Change** |
| --- | --- | --- |
| 0 | 5 & 6 | We significantly revised the end of the introduction to specifically describe the purpose of our work, and to contextualize that purpose within a wider body of literature. Similarly, we clarify the distinction between hypothesis-driven research and real-world, discovery-based research. |
| 1 | 3 | STEM and MOOCs have been spelled out in the key words |
| 2 | 3 & 18 | The number has stayed on p. 9, but the comparison with prior research and courses has moved to the discussion on p. 18. |
| 3 | 6 | The opening line of methods has been modified to specifically indicate the the two courses, an introductory and advanced MOOC will be described in methods under “Course structure and logistics” |
| 4 | 10 | These percentages have been added to p. 10. In addition, the manuscript has been corrected to refer to S6 Table, not S6 Fig. |
| 5 | 10 | The threshold is now defined on p. 11, an oversight on our part. |
| 6 | 12 | On p. 12 the faculty number was corrected to 20%, not 2%. In addition, the other category percentages and a description were added. |
| 7 | 13 | “willingness” has been changed to lower case on p. 13, apologies for the typo. |
| 8 | 17 | The section on "Open educational resource engagement" has been moved to the discussion on p. 17 |
| 9 | 71 | As recommended, an opening paragraph has been added on p. 17 to the discussion that reiterates the context of this work as discovery-based research, explicates the guiding questions and specifies the key findings. |
| 10 | 3 & 4 | The first instance of ‘postdocs’ on p. 3 is expanded, and explicated as ‘postdocs’ hereafter. The first instance of MOOCs on p. 4 in the manuscript had already been expanded and attached to the commonplace acronym. |

**Comments by reviewer with itemized changes:**

Reviewer #1:

Reviewer #1: Excellent work on revisions, this manuscript is ready for publication. I verify that all required questions have been answered and that all responses meet formatting specifications.

*Thank you!*

Reviewer #3:

“Thank you for your revisions. These have improved the readability of your paper. Unfortunately, despite changes your manuscript remains somewhat challenging to follow. I would suspect that your decision to pursue "guiding questions" rather than "formal hypotheses" significantly impairs the readability of the paper. This is because your key message(s) are not clearly stated making it particularly hard to define the purpose of the paper and key messages. To be useful to the reader, the authors must clearly state their purposes and ensure that the key take away points are clearly stated for the reader at the start of the discussion and at the conclusion of reading the paper.”

*Change #0: We significantly revised the end of the introduction to specifically describe the purpose of our work, and to contextualize that purpose within a wider body of literature. Similarly, we clarify the distinction between hypothesis-driven research and real-world, discovery-based research that we intentionally pursue.*

“Abbreviations STEM and MOOC should not be used in keywords - this has not been addressed.”

*Change #1: STEM and MOOCs have been spelled out in the key words*

“Line 205 : The average introductory course completion rate of 11.5% overall is more than double the rate reported for other non-professional and non-degree MOOCs [34, 35, 25]. Belongs in discussion as this is not your results but rather a discussion of your results”

*Change #2: The number has stayed on p. 9, but the comparison with prior research and courses has moved to the discussion on p. 18.*

“Line 207: You report an "advanced MOOC" but this is never / not clearly defined in your methodology. Please specify in the methods that there are 2 MOOCs being evaluated and how these differ.”

*Change #3: The opening line of methods has been modified to specifically indicate the two courses, an “introductory” and “advanced” MOOC will be described in methods under “Course structure and logistics”*

*Additional response to reviewer #3: The methods section on p. 6 and 7 describe the detailed differences between the introductory and advanced MOOCs.*

“Line 215: "substantially outranked achievement oriented goals including “earn credentials for my CV” and “earn a statement of accomplishment.”" but the percentaged [sic] for both these are not specified. They should be specified in the results to support your statement that these were "substantially" different”

*Change #4: These percentages have been added to p. 10. In addition, the manuscript has been corrected to refer to S6 Table, not S6 Fig.*

“Line 233 The "threshold" for "drop-offs" is undefined in the methodology”

*Change #5: The threshold is now defined on p. 11, an oversight on our part.*

“Line 252-3 : "Pre- and post-course respondents were largely PhDs and postdocs (50% pre-course and 58% post course) with faculty an additional 2%." Who were the other 48%?”

*Change #6: On p. 12 the faculty number was corrected to 20%, not 2%. In addition, the other category percentages and a description were added.*

“Line 284: "Willingness" should be lower case”

*Change #7: “willingness” has been changed to lower case on p. 13*

“Line 340-352: Unclear why this "Open educational resource engagement" section is in the results section. Not sure what results are being shared here. Does this belong in the discussion?”

*Change #8: The section on "Open educational resource engagement" has been moved to the discussion on p. 17 per the recommendation.*

“Discussion: In your opening paragraph you need to sumarize [sic] what your key findings were. Based on your format of using guiding questions, this should be a 3-4 line paragraph which summarizes clearly what your take key [sic] results/findings of your study are. Tyipcally [sic] this would be a place where you would discuss your hypotheses and if they were proven or disproven.”

*Change #9: As recommended, an opening paragraph has now been added on p. 17 to the discussion that reiterates the context of this work as discovery-based research, explicates the guiding questions and specifies the key findings.*

Abbreviations:

- postdocs - should be expanded

- MOOCs - needs to be defined in body of manuscript not just abstract

*Change #10: The first instance of ‘postdocs’ on p. 3 is expanded, and explicated as ‘postdocs’ hereafter. The first instance of MOOCs on p. 4 in the manuscript had already been expanded and attached to the commonplace acronym.*

*We hope these revisions now meet with the expectations of reviewer #3, the Associate Editor, and Editor.*
